# Retinitis Pigmentosa Due to Rp1 Biallelic Variants

**DOI:** 10.1038/s41598-020-58243-9

**Published:** 2020-01-31

**Authors:** Rita Sousa Silva, Mariana Vallim Salles, Fabiana Louise Motta, Juliana Maria Ferraz Sallum

**Affiliations:** 1 Department of Ophthalmology, Ophthalmology Institute Dr. Gama Pinto, Lisbon, Portugal; 2 Department of Ophthalmology, Federal University of Sao Paulo, Sao Paulo, Brazil

**Keywords:** Epidemiology, Epidemiology, Genetics research, Genetics research

## Abstract

In the present study, we screened 529 Brazilian individuals affected by inherited retinal disorders. A total of seven unrelated and nonsyndromic patients with RP1 biallelic variants (OMIM # 180100) were diagnosed in our centre and included in the study. They had classic retinitis pigmentosa with diagnosis at the first decade of life. The visual acuities were severely affected at a young age. The fundus aspects were similar among all patients. An atrophic ring was present around the fovea in several cases. All patients had molecular diagnosis, with six different RP1 variants. This study reports two new pathogenic variants - two frameshift duplications (c.1234dupA p.Met412Asnfs*7 and c.1265dupC p.Ala423Cysfs*2) and reinforces other four known pathogenic variants – two frameshift deletions (c.469delG p.Val157Trpfs*16 and c.3843delT p.Pro1282Leufs*12) and two stop gain mutations (c.1186 C > T p.Arg396* and c.1625C > G p.Ser542*). These findings broaden the spectrum of RP1 variants. This study also reviewed the fundus characteristics that clinically could raise the hypothesis of a retinitis pigmentosa due to RP1 gene. It is worthwhile to try to identify the disease-causing variants in each patient since it can provide prognostic information and be useful in genetic consultation and diagnosis in the future.

## Introduction

Retinitis pigmentosa (RP; Online Mendelian Inheritance in Man - OMIM #268000), refers to a clinically and genetically heterogeneous group of progressive inherited retinal disorders (IRD) that result in retinal degeneration, affecting 1 in 4000 people^1–3^. There is tremendous heterogeneity according to the age of onset, progression, retinal appearance and visual outcome^4,5^. The disease is characterized by nyctalopia and progressive visual field loss due to primary rod dysfunction. In most cases, it also courses with dyschromatopsia and progressive loss of central vision owing to secondary cone degeneration^1,3^. On examination, patients have a classic fundus appearance with ‘bone spicules’ - dark clumps of pigment in the midperiphery and perivenous areas - and attenuated retinal vessels, cystoid macular oedema and waxy optic disc pallor^4^.

Most commonly, RP is inherited as a Mendelian trait but its genetics is more complex than once expected^3^. The disease can be divided into 3 patterns of inheritance: autosomal recessive (ArRP) (50–60%), autosomal dominant (AdRP) (30–40%), and X linked RP (xLRP) (5–15%)^3,6^, and up to 50% represent isolated cases due to the absence of family history, simplex RP (sRP)^4^. To date, 93 genes of RP were mapped and identified - 29 AdRP, 61 ArRP and 3 xLRP, (until February 5, 2018, https://sph.uth.edu/retnet/sum-dis.htm#C-complex). Although most cases follow the most common forms of classical Mendelian inheritance patterns, these are not exclusive in this disease.

RP1 (OMIM #180100) is one of many genes analysed in molecular diagnosis for IRD. Because of its response to *in vivo* retinal oxygen levels, this protein was initially named oxygen-regulated protein-1. Afterwards, it was found that mutations in this gene cause autosomal dominant RP, so the protein was subsequently named RP1^7^.

RP1 is a photoreceptor-specific protein expressed in both rods and cones, and appears to be important for the correct orientation and stacking of discs during outer segment morphogenesis^3,5^. RP1 is located on chromosome 8q12.1, contains 4 exons and encodes for a 2156 amino acid-long protein that is located in the connecting cilia and axoneme of the photoreceptors. The N-terminal 233 amino-acid residue of the protein is where the microtubule-binding domain of doublecortin (DCX) is situated^4,5,8^. DCX domain is essential in long-term survival of photoreceptors since it binds and stabilizes microtubules and controls ciliary length^4^. Similarly to other IRD proteins, the function of RP1 protein is still not fully understood.

RP1 gene variants can cause AdRP and ArRP disease and include single nucleotide substitutions that produce a premature stop codon or insertions/deletions resulting in a truncated protein of approximately 50–70% of its full length, which can result in severe early-onset RP^8^.

Although the RP1 gene has four exons, exon 4 encodes for more than 85% of the expressed protein. Most known RP1 gene variants are located in exon 4^4^. This retrospective study aimed to describe the phenotype and the genotype of Brazilian patients with RP due to RP1 gene biallelic variants.

## Results

### Clinical data

Clinical data is summarized in Table 1. Among the 529 medical records of Brazilian patients analysed, seven unrelated and nonsyndromic RP patients with RP1 variants were selected. Three were female and four were male, with mean age at diagnosis of 8,6 ± 4,4 years (min. 2, max. 16). They were all isolated cases, suggesting an autosomal recessive pattern or autosomal dominant de novo inheritance pattern. Three declared consanguineous parents (patient 5, 6 and 7).

**Table 1:** Clinical data of *RP1* patients.

PATIENT	GENDER	ACTUAL AGE	AGE AT TIME OF DIAGNOSIS	MEDICAL AND OPHTHALMOLOGICAL HISTORY	REVELANT FAMILY HISTORY	SYMPTOMS AT TIME OF DIAGNOSIS	BCVA (RE; LE)	FUNDUS EXAMINATION
1	F	31	10	OS cataract surgery Intermittent exotropia Recent nystagmus	None	Nyctalopia and reduced visual field	20/200; <20/400	NBV, BS in the periphery and perifoveal pigmentation with a sketch of atrophic macular ring
2	M	28	8	Myopia^+^	Progressive myopia	Nyctalopia and reduced visual field	5/400; 5/400	NBV, little RPE changes in the periphery and perifoveal pigmentation with macular atrophy
3	M	63	16	Bilateral cataract surgery	Progressive myopia and RP	Nyctalopia and reduced visual field	LP; LP	NBV, BS in the periphery and perifoveal pigmentation with atrophic macular ring
4	F	24	7	Myopia^+^	None	Nyctalopia and reduced visual field	CF; 20/150	NBV, widespread RPE changes with BS in the periphery, perifoveal pigmentation with macular atrophy, pale optic discs
5	M	14	2	High myopia^+^	None	Nyctalopia, reduced visual acuity and visual field	20/70; 20/20	NBV, little RPE changes in the periphery, little perifoveal pigmentation without atrophy, well coloured optic nerve heads
6	M	30	6	Irritable Bowel Syndrome	None	Reduced visual acuity	HM; LP	NBV, BS in the periphery and perifoveal pigmentation with atrophic macular ring
7	F	31	11	Glaucoma	Werding-Hoffmann Syndrome	Reduced visual acuity	<20/400; <20/400	NBV, BS in the periphery and perifoveal pigmentation with atrophic macular ring

^+^3/7 patients had myopia. The patient with high myopia had good BCVA.

Light perception (LP); count fingers (CF); hand movement (HM); narrowed blood vessels (NBV); bone spicules (BS).

**Figure 1:**
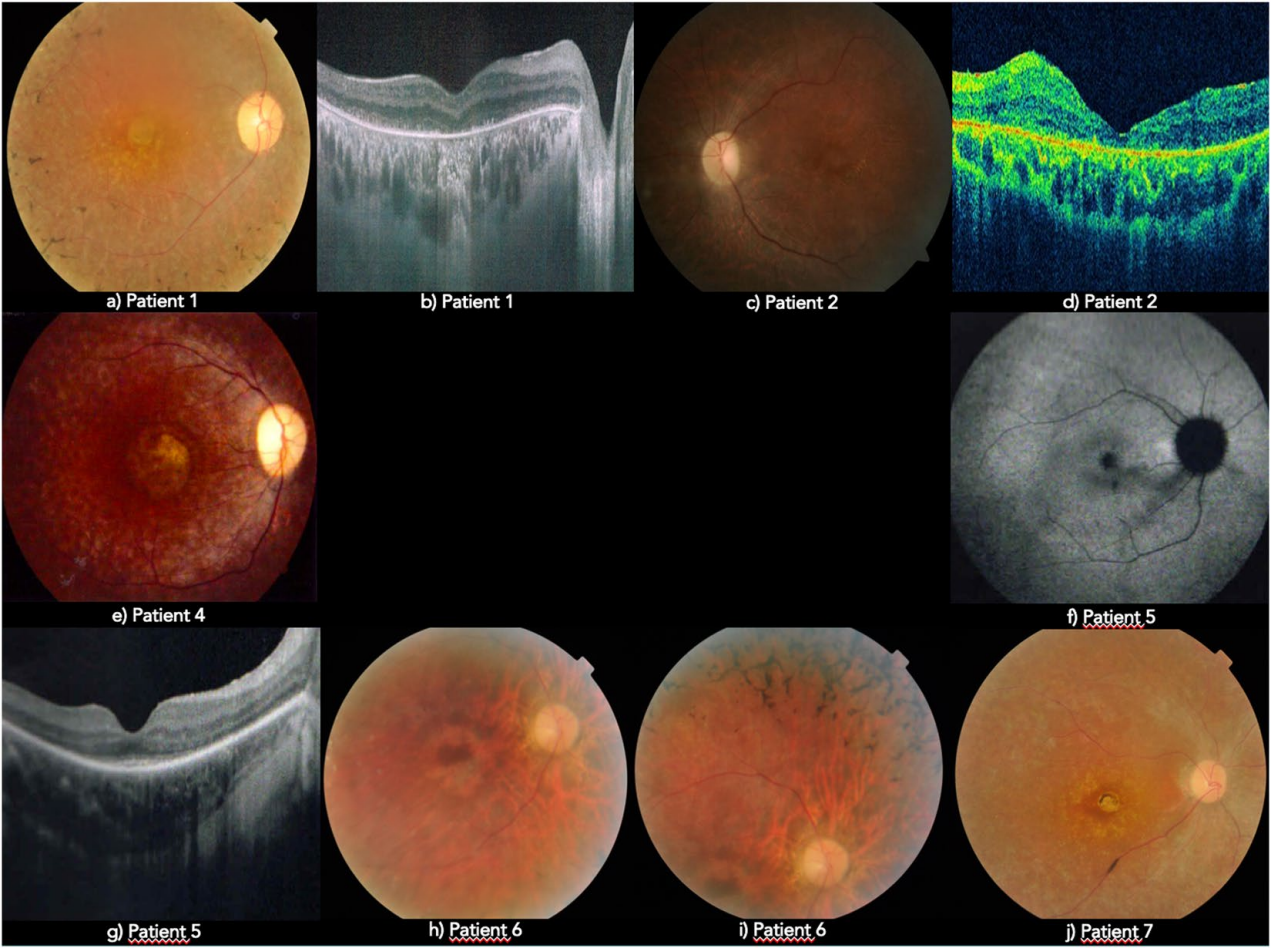
Fundus appearance from *RP1* patients: (**a**,**c**,**e**,**h**,**i**,**j**) - CFP; (**f**) – FAF; (**b**,**d**,**g**) - OCT.

Age at time of examination and diagnosis ranged between 2 and 32 years. Only one patient was not diagnosed during childhood (patient 3). Symptoms at time of diagnosis were mostly based on night blindness and visual field defects. Visual acuities were severely affected from a young age. Some patients had moderate myopia and one had high myopia of -9 dioptres (patient 5). Fundus changes were similar between patients, with narrow blood vessels, pale optic disc and mild to widespread osteoclast pigmentation, which can be seen in CFP (Fig. 1). Almost all patients (except patient 5) presented with a certain degree of macular atrophy, with some showing a well-defined atrophic macular ring (patient 3, 6 and 7), as documented in the OCT. Due to the retrospective nature of this study, there are specific data which we did not have access to. Certain documents are in the patients’ possession, thus missing from their clinical records.

### Genetic findings

**Figure 2:**
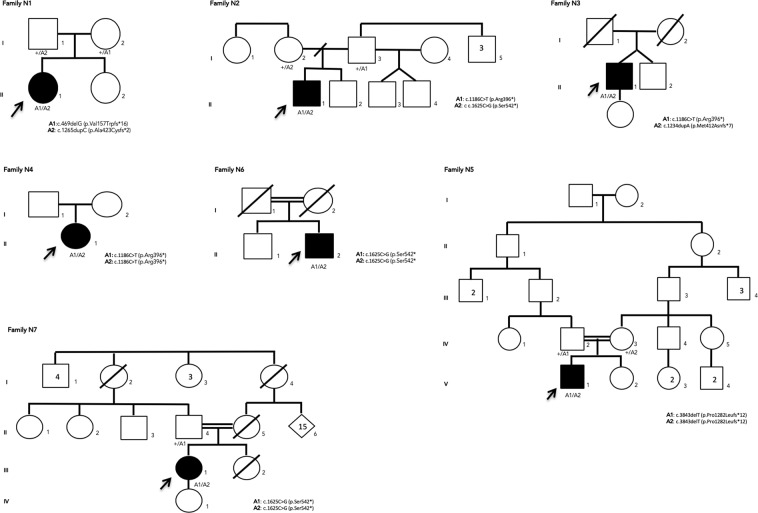
Pedigrees of seven families with RP1 mutations. Affected and unaffected individuals are represented by shapes filled with black and white colours, respectively. Men and women are indicated by squares and circles, respectively. Index subjects are marked by ↑. Consanguinity is marked by a double horizontal line. Normal and mutated alleles for each individual are labelled (+) and (M) respectively.

Table 2 describes the genotypes of those patients. All patients showed two RP1 pathogenic variants in their molecular test. Segregation analysis revealed that each variant was inherited from a different progenitor, except in families F3, F4 e F6 since relatives’ samples were not available (Fig. 2). The variants found in these subjects are preferably located in exon 4, with the exception of c.469delG (located in exon 2). Three patients were compound heterozygotes (patients 1, 2 and 3) and the other four were homozygotes (patients 4, 5, 6 and 7). Furthermore, three of the homozygotic patients were offspring from a consanguineous marriage (families F5, F6 and F7).

**Table 2:** Genetic data of *RP1* patients.

PATIENT	ALLELE 1				ALLELE 2			
	NUCLEOTIDE CHANGE	PROTEIN CHANGE	EXON	EFFECT	NUCLEOTIDE CHANGE	PROTEIN CHANGE	EXON	EFFECT
1	c.469delG	p.Val157Trpfs*16	2	FS	c.1265dupC	p.Ala423Cysfs*2^**†**^	4	FS
2	c.1186 C > T	p.Arg396*	4	SG	c.1625C > G	p.Ser542*	4	SG
3	c.1186 C > T	p.Arg396*	4	SG	c.1234dupA	p.Met412Asnfs*7^†^	4	FS
4	c.1186 C > T	p.Arg396*	4	SG	c.1186 C > T	p.Arg396*	4	SG
5	c.3843delT	p.Pro1282Leufs*12	4	FS	C.3843delT	p.Pro1282Leufs*12	4	FS
6	c.1625C > G	p.Ser542*	4	SG	c.1625C > G	p.Ser542*	4	SG
7	c.1625C > G	p.Ser542*	4	SG	c.1625C > G	p.Ser542*	4	SG

Patient 4, 5, 6 and 7 are homozygotes. (FS – frameshift; SG – stop gain; M – missense; ^†^NOVEL Variants).

Two novel frameshift duplications were found, c.1234dupA (p.Met412Asnfs*7) and c.1265dupC (p.Ala423Cysfs*2). None of them were found in Human Gene Mutation Database (HGMD) and ClinVar; they were found in low frequencies in Genome Aggregation Database (gnomAD) (the first with a frequency of 4.064 × 10–6 and the second with a frequency of 8.126 × 10-6). In this study, we also reinforced the other four known variants as disease-causing mutations: two frameshift deletions c.469delG (p.Val157Trpfs*16) and c.3843delT (p.Pro1282Leufs*12) and two stop gain c.1186 C > T (p.Arg396*) and c.1625C > G (p.Ser542*) - Table 3. Within this small cohort of RP1 patients, c.1625C > G (p.Ser542*) and c.1186 C > T (p.Arg396*) were the most frequent variants, accounting for 35,7% and 28,6% of analysed alleles respectively.

**Table 3:** Variants data from *RP1* patients.

*RP1* VARIANTS	HGMD ACCESSION	REFERENCE	ALLELE FREQUENCY IN THIS STUDY
c.469delG (p.Val157Trpfs*16)	CD1510116	Lafont *et al*.^5^	1/16
c.1186 C > T (p.Arg396*)	CM1312698	Bocquet *et al*.^10^	4/16
c.1234dupA (p.Met412Asnfs*7)		NOT REPORTED	1/16
c.1265dupC (p.Ala423Cysfs*2)		NOT REPORTED	1/16
c.1625C > G (p.Ser542*)	CM1211361	Avila-Fernandez *et al*.^4^ Wang *et al*.^17^ El Shamieh *et al*.^3^ Ezquerra-Inchausti *et al*.^18^ Pérez-Carro *et al*.^19^	5/16
C.3843delT (p.Pro1282Leufs*12)	CD1312799	Eisenberger *et al*.^11,17,20,21^	2/16

## Discussion

Pathogenic variants in the RP1 gene often lead to the production of a truncated protein^4^. As seen in this study, all 14 alleles analysed contain frameshift or nonsense variants. In addition, all RP1 patients in this study had two pathogenic variants in trans, this indicates that the disease followed an autosomal recessive inheritance pattern. The disease course of RP1 variants is known to be much more severe in autosomal recessive than in autosomal dominant cases, with a dramatic decrease in the visual acuity and tubular vision by the end of the third decade^5^.

All patients had premature stop codon formation in both alleles, presenting a severe phenotype with onset before adulthood; most of them were diagnosed before adolescence (patient 3 was the exception). RP1 truncating mutations can be classified in four categories depending on their location (according to Chen *et al*.)^2,9^ This classification is also directly related to the pattern of inheritance. Among all the variants with premature stop codon formation found in this study, only p.Ser542* did not follow the proposed rule by Chen and colleagues. Regarding location, this variant belongs to class II (mutations between amino acid 500 and 1053) so it should cause an AdRP, however in this study and in others it causes an autosomal recessive disease^3,4^. Other variants located between amino acid 500 and 1053 (class II) also did not follow the rule of causing autosomal dominant RP such as, p.Ser574Cysfs*7, p.Ser676Ilefs*22, p.Arg793Glufs*55, p.Asp799* and p.Asn949Lysfs*32^2,3^.

Patients 2, 6 and 7 showed variants involving p.Ser542*, for which patient 6 and 7 were homozygous. (see Table 2). p.Ser542* seems to be a common recessive founder mutation in some cohorts of Spanish patients^4^. As compared with other reported cases, all patients shared a common phenotype characterized by early-onset RP with secondary macular involvement, even with different ArRP mutations in the RP1 gene^3,4^. Our clinical study showed that all of the affected patients had severe RP with decreased visual acuity, bilateral macular involvement with an abnormal foveal reflex, dark perifoveal area and macular atrophy. While patient 2 and 7 had few pigment deposits, patient 6 had widespread bone spicules. Patient 2 was the only one in this subgroup with myopia.

Variant p.Arg396* was found in patients 2, 3 and 4. All patients with this variant, including patients from other studies^10^, share a common phenotype characterized by severe early-onset RP with secondary macular involvement and few bone spicules in the periphery. Patient 4 was the only homozygote and also the only one from this subgroup without myopia. Patient 3 as a compound heterozygote, also had the variant p.Met412Asnfs*7, which has never been described before.

Patient 1 had p.Ala423Cysfs*2, not described in the literature, and p.Val157Trpfs*16 variants. Similarly to other carriers of premature stop codons, this patient underwent dramatic decrease in visual acuity and tubular vision in her third decade of life. Her fundus showed typical bone spicules covering the periphery, narrowing of retinal vessels and moderate macular involvement with atrophy, similarly to other study samples of p.Val157Trpfs*16 variant. However, unlike other patients in this group, patient 1 was not myopic^6^.

p.Pro1282Leufs*12 variant was described only once before, in a 37-year-old Turkish woman homozygous for this variant^11^. Patient 5 also had p.Pro1282Leufs*12 variant in a homozygous state. Since patient 5 was the youngest patient in this study group, he only had a small decrease in visual acuity of his right eye, little bone spicules in the periphery and no macular atrophy. Nevertheless, he already had macular involvement with perifoveal pigmentation.

As previously reported, RP1 can lead to different patterns of disease, with significant superior and temporal visual field defects and bone spicules predominantly in the inferior fundus^12^. This disposition was not found in this case series. Macular atrophy was found in almost all patients, in addition to the typical RP fundus aspects highlighted previously. The most frequent RP1 mutation reported is c.2029 C > T (p.Arg677*), that causes AdRP with a high variability of disease expression^2,4,7^. Due to the fact that it causes only autosomal dominant RP, it was expected that this variant would not be found in this cohort of autosomal recessive RP. his series represents simplex cases with severe early-onset disease, including markedly decreased visual acuity and severe visual field defects by the end of the third decade of life. This study reinforces the notion that patients with mutations in both RP1 alleles present severe early-onset RP with macular atrophy compared to patients with only one pathogenic variant who present late-onset and slowly progressing forms of the disease^2,13^. In addition, there seems to be an association between myopia and this gene-specific form of RP^6^.

The outer retina is susceptible to many insults, which makes it a vulnerable target, leading to retinal pigment epithelium apoptosis. Retinal remodelling starts at mid-periphery and progresses to the posterior pole and central retina. The exact mechanism of cell death in RP is still unknown. However, retinal oxygen modulation is believed to have a role in the pathogenesis of retinal neovascularization^14^. Oxidative stress is also known to play a major role in this process^15^. This will be the object of further research in the Phase 1 FIGHT-RP1 STUDY at Johns Hopkins University, where the role of orally administered N-Acetylcysteine (NAC) in the promotion of survival and maintenance of cone function in patients with RP will be explored^16^.

## Methods

This retrospective study reviewed 529 medical records of Brazilian patients referred to the Universidade Federal de São Paulo and Instituto de Genética Ocular in São Paulo, Brazil, between January 2006 and December 2017 and who underwent genetic testing for IRD. The medical record of seven unrelated Brazilian patients with RP1 variants were selected. Clinical diagnosis, according to patient notes, was made based on history and physical examination. In some patients, assessment was complemented with multimodal imaging, such as colour fundus photography (CFP), optical coherence tomography (OCT), fundus autofluorescence (FAF), visual field (VF) and electroretinogram (ERG) testing. The genetic tests performed were sequencing of hereditary retinopathies panel (226 genes) using the Illumina HiSeq platform, conducted by a Brazilian private commercial laboratory. Mutation segregation analysis was carried out where relatives’ samples were available.

The variants identified were classified for the pathogenicity as follows: (1) variants that have already been reported as disease-causing in the literature, (2) variants leading to protein loss-of-function, (3) variants absent in population genetic databases, (4) variants present in other affected patients, and (5) variants that have not been reported in the literature, but are predicted to be disease-causing by in silico analysis. The databases used were HGMD, ExAC, GnomAD, ClinVar and ESP. The study was approved by the Ethics Committee in Research of Universidade Federal de São Paulo (CEP:0415/2016). Written informed consents were obtained after explanation of the nature of the study. All procedures were conducted in accordance to the Declaration of Helsinki.

## Supplementary Information

Supplementary Information.

## Data Availability

The datasets generated during the current study are available from the corresponding author on reasonable request.
