# Supplementary material for: Retinitis Pigmentosa Due to Rp1 Biallelic Variants
Source: Sci Rep. 2020 Jan 31;10:1603. doi: 10.1038/s41598-020-58243-9 (PMC6994576; doi:10.1038/s41598-020-58243-9)

RETINITIS PIGMENTOSA DUE TO RP1 BIALLELIC VARIANTS

Rita Sousa Silva^1^, Mariana Vallim Salles^2^, Fabiana Louise Motta^2^, Juliana Maria Ferraz Sallum^2*^

Affiliations

1. Department of Ophthalmology, Ophthalmology Institute Dr. Gama Pinto, Lisbon, Portugal

Rita Sousa Silva

1. Department of Ophthalmology, Federal University of Sao Paulo, Sao Paulo, Brazil

Mariana Vallim Salles, Fabiana Louise Motta & Juliana Maria Ferraz Sallum

**SUPPLEMENTARY INFORMATION**

Unaffected progenitors with normal retinographies


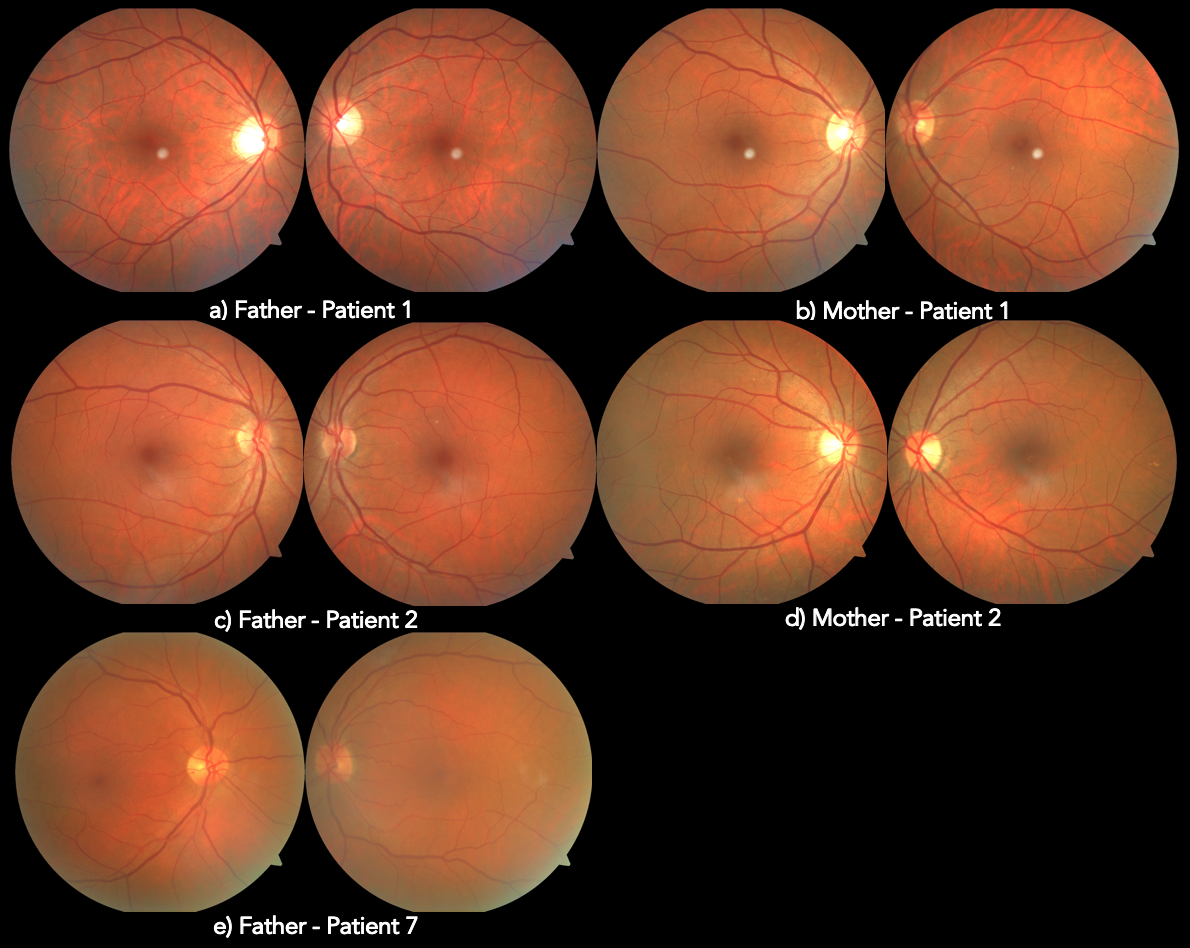

Supplement: Supplementary file 1 — Supplementary Information. [file 41598_2020_58243_MOESM1_ESM.docx]
